# Phaeohyphomycosis Caused by *Pleurostomophora richardsiae*: A Case Report

**DOI:** 10.1007/s11046-023-00764-8

**Published:** 2023-07-01

**Authors:** Yijun Mo, Yao Lu, Bei Wang, Jingjing Wang, Yueru Tian, Hong Liu

**Affiliations:** 1 Department of Laboratory Medicine, Ningbo First Hospital, Ningbo Hospital of Ningbo University, Ningbo, 315010 Zhejiang China; 2 Department of Laboratory Medicine, Huashan Hospital, Fudan University, Shanghai, 200040 China

**Fig. 1:**
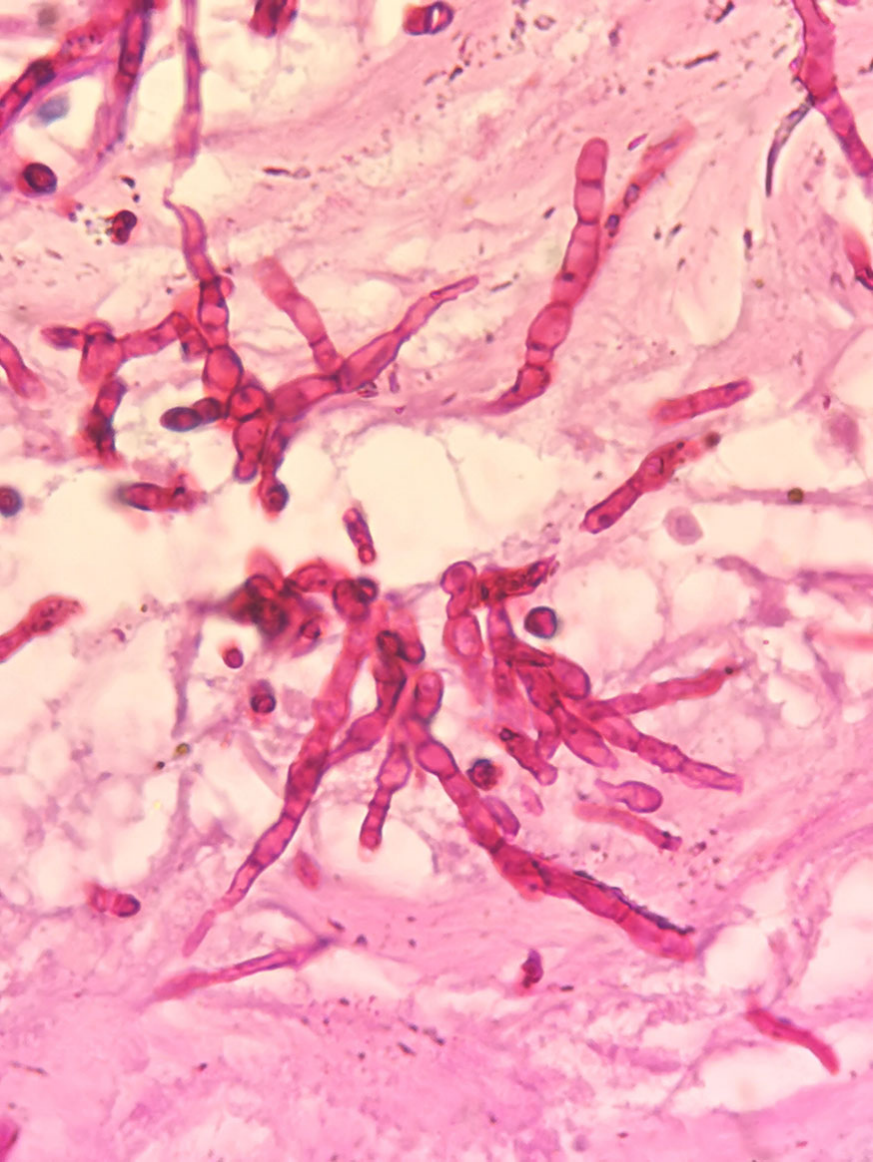
PAS Stain showing conidia and fungal hyphae

**Fig. 2:**
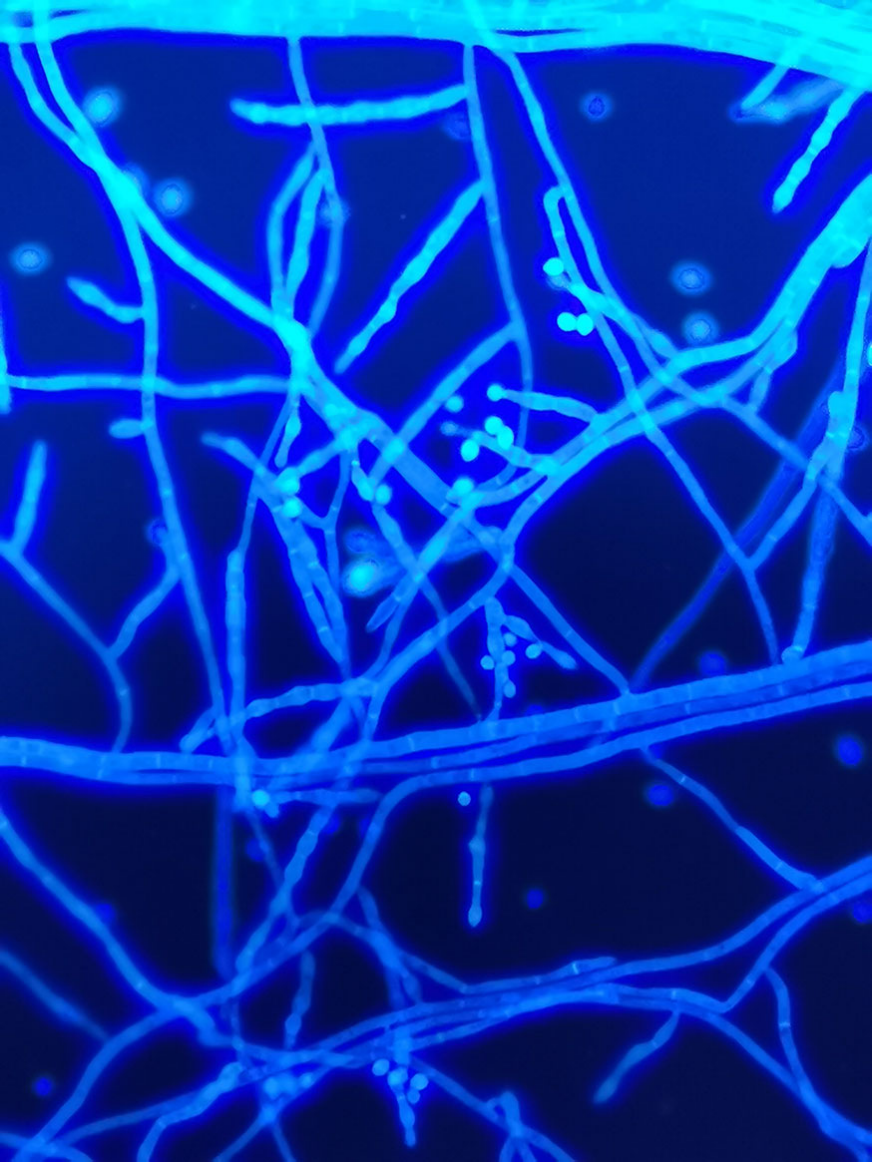
Fluorescence Stain(×400)showing  Single bottle stem visible at the tip or side of the hyphae

**Fig. 3:**
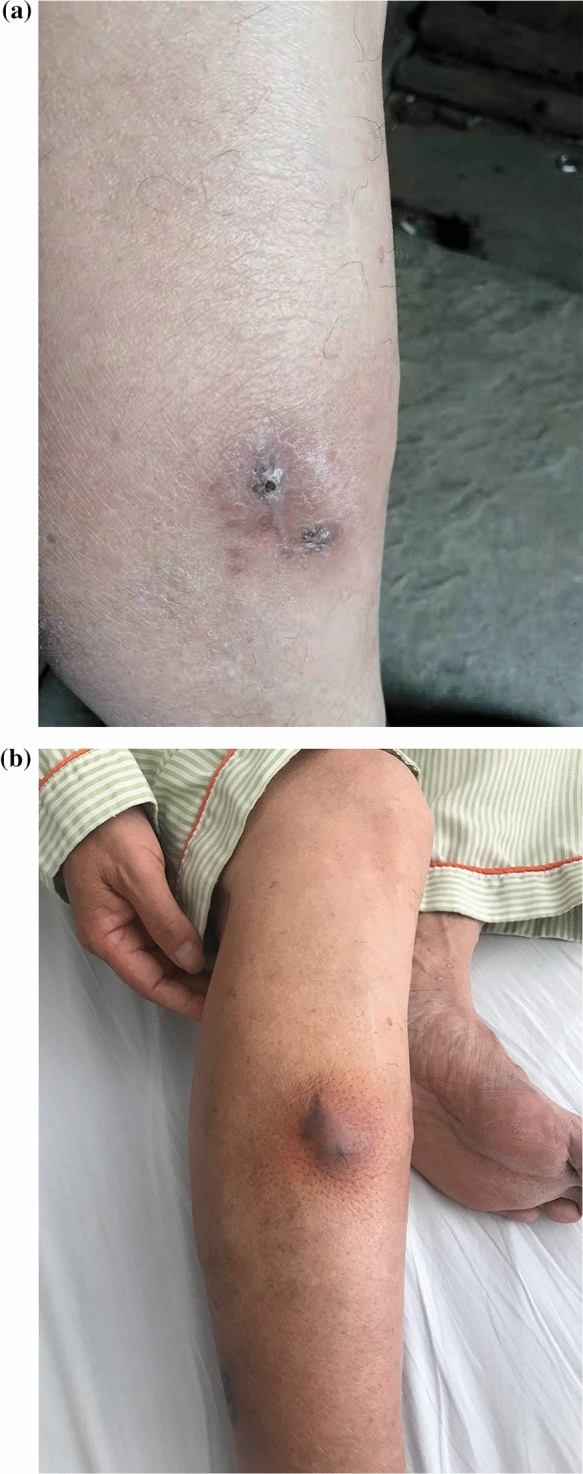
(**a**) Clinical photograph of patient’s leg after treatment. (**b**) Clinical photograph of patient’s leg before treatment

*Pleurostomophora richardsiae* (*P. richardsiae*) is one of many dematiaceous (darkly pigmented or black) fungi that can be isolated from the soil and vegetation in tropical climates. In China, there have been no reported cases of *P. richardsiae* infections in humans until now. Here, we report the rare fungal infection in a 67-year-old man. In the second half of 2017, the patient developed multiple peanut-sized nodules on his right lower leg with no apparent cause. The nodules showed no obvious redness, swelling, pain, or septic ulceration, and disappeared after the application of saline compresses by the patient. The patient suffered no chills, fever, cough, sputum, or other uncomfortable symptoms. In early 2018, the nodules on the right lower leg recurred and gradually increased in number, with some accompanied by suppurative discharge and tenderness upon palpation. The patient’s skin temperature was not elevated, and the nodules healed by themselves. There was some slight edema in the right lower limb after activity, which was relieved by rest, with neither numbness nor pain. In January 2021, the patient visited a People’s Hospital in Shanghai. Purulent material was drained from the nodules for a tissue pathology analysis, which revealed granulomatous inflammation with small abscesses, local granulomatous tissue hyperplasia with interstitial fibrosis, and hemosiderin deposition. An immunohistochemical analysis excluded the possibility of a tumor. The patient was treated with cephalosporin antibacterial drugs, but with no obvious improvement. One of the nodules became swollen and tender upon palpation, prompting the patient to seek treatment at our hospital. After admission, the patient underwent a dermatological biopsy. Samples were sent for microscopic evaluation, prolonged culture, tissue pathology, and high-throughput pathogen metagenomic sequencing (PMseq™). A few days later, the pathological analysis revealed local radial clusters, and with periodic acid-Schiff (PAS) staining, and PAS-positive clusters were seen in the dermis (Fig. 1). The fungus was also cultured from living tissue in the hospital’s mycology department, and was identified as *P. richardsiae* by morphological observation (Fig. 2) and DNA sequencing analyses of the internal transcribed spacer (ITS) and the 18S rRNA gene. Meanwhile, the patient mentioned that he walked barefoot in the fields and fished in the local river. There is no standard treatment protocol for phaeohyphomycosis. In China, the treatment is mainly based on systematic antifungal treatment, and itraconazole is the most commonly used drug. Finally, itraconazole was administered orally at 200 mg twice daily. Figure 3 shows the patient’s skin lesion on October 23, which was much improved. *P. richardsiae* infection is rare in humans. Infected patients often pay no attention to the skin lesions, and usually seek medical treatment only after several months or even years. Most patients have difficulty recalling a history of trauma, and healthcare professionals also tend to overlook rare fungal infections. An analysis of clinical manifestations, mycological testing, and histopathology are required for its diagnosis. Genetic sequencing and other molecular biology techniques should be combined with traditional mycological and pathological examinations to identify the causative organism.

